# Pedunculated uterine leiomyoma mimicking abdominal mass: a case report

**DOI:** 10.1186/1757-1626-1-315

**Published:** 2008-11-17

**Authors:** Athina Spanoudaki, Anastasia Oikonomou, Krasimira Dimitrova, Panos Prassopoulos

**Affiliations:** 1 Department of Radiology, University Hospital of Alexandroupolis, Democritus University of Thrace, Greece

## Abstract

A 48-year-old woman presented with abdominal fullness and a palpable "mass" in the left lower quadrant. Ultrasonography showed a large, rounded, hypoechoic mass. Contrast-enhanced helical CT of the abdomen demonstrated a well-circumscribed, heterogeneously but vividly enhancing mass. The uterus had a leiomyomatous configuration on CT. Uterus and mass revealed the same enhancing pattern. Thin section CT revealed a long, thin stalk connecting the mass with the body of the uterus. Surgical removal of both uterus and the mass confirmed the diagnosis of a pedunculated subserosal leiomyoma originating from a leiomyomatous uterus.

## Case presentation

A 48-year-old woman presented with a sensation of fullness in the abdomen and a palpable mass in the left lower quadrant of the abdomen. She reported no changes in her menstrual cycle or bowel habits.

**Figure 1:**
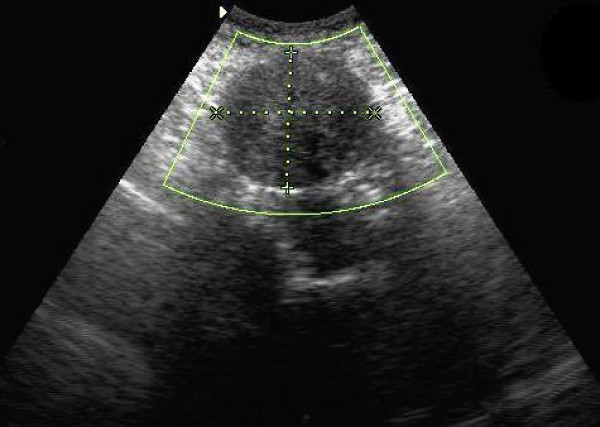
**Ultrasound of the abdomen, left lower quadrant**. US shows a large, rounded, well-defined hypoechoic mass in the left lower quadrant abdomen.

**Figure 2:**
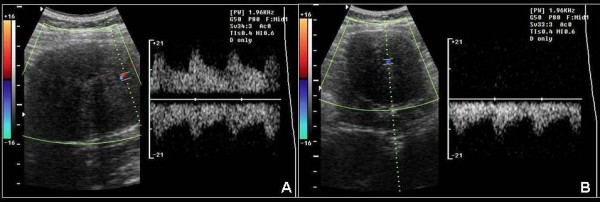
**A, B. Color Doppler ultrasound of the abdomen**. Color Doppler US detects minimal flow within the mass.

**Figure 3:**
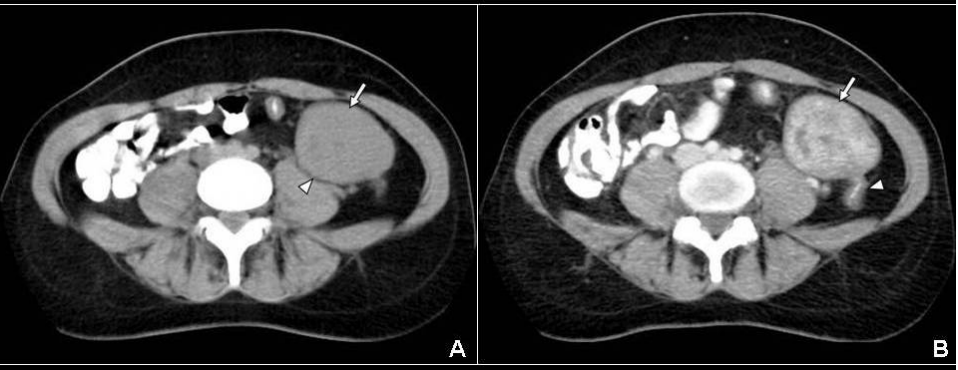
**A, B. A: Axial non-enhanced CT scan at the level of aortic bifurcation, demonstrates a rounded, well-circumscribed, mass of soft tissue density (arrow) in the left lower quadrant abdomen.** The mass abuts the left psoas muscle (arrowhead) showing an intact interface with it. B: Contrast-enhanced CT scan at the same level with figure 3A, exhibits marked heterogeneous enhancement of the mass (arrow). Note the contrast-filled bowel loop being in direct proximity with the posterior surface of the mass (arrowhead).

**Figure 4:**
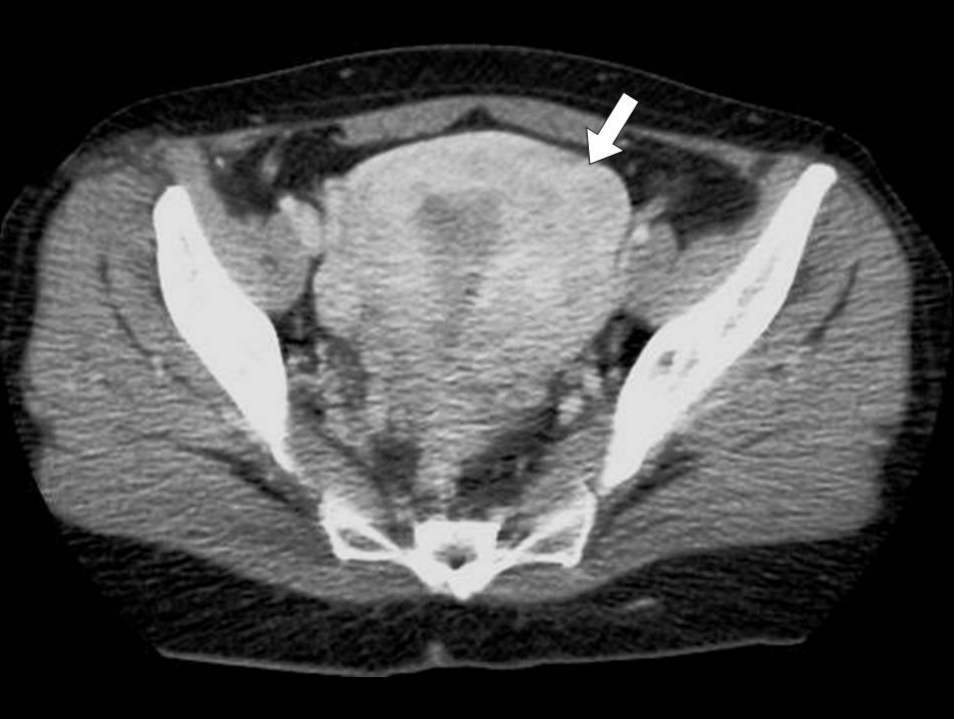
**Contrast-enhanced axial computed tomography scan of the pelvis**. Contrast-enhanced axial computed tomography scan of the pelvis demonstrates an enlarged uterus with deformed contour (arrow), consistent with leiomyomatous uterus. The uterus enhances heterogeneously and vividly in a similar way compared to the previously described mass in the left lower quadrant (figure 3B).

**Figure 5:**
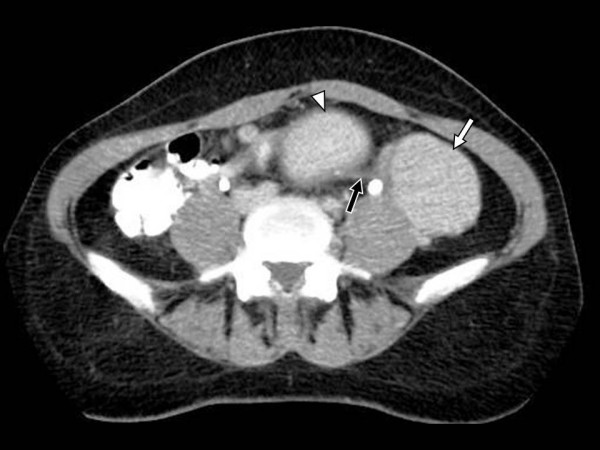
**Thin section contrast-enhanced computed tomography of the lower abdomen**. Thin section contrast-enhanced CT scan targeted at the level of the mass reveals a thin stalk (black arrow) connecting the mass (white arrow) with the upper left body of the uterus (arrowhead).

Physical examination revealed a large palpable, relatively mobile, nontender mass in the left lower quadrant of the abdomen. Laboratory test values were within normal limits. Ultrasonography (US) showed a large, rounded, homogeneous, relatively hypoechoic mass, measuring 5 × 6 cm (Fig 1). Color Doppler US detected minimal vascular flow within the mass (Fig 2). Contrast-enhanced helical CT of the abdomen demonstrated a well-circumscribed, heterogeneously but vividly enhancing mass in the left lower abdominal quadrant (Fig 3). The mass was in close relationship – but with intact interface – with the left psoas muscle and contrast-filled bowel loops. The uterus was enlarged with a deformed uterine contour consistent with leiomyomatous uterus (Fig 4). The uterus and the left lower quadrant mass had the same heterogeneous, yet vivid enhancing pattern. Thin section CT targeted at the mass, revealed a 2 cm-long, thin stalk connecting the mass with the upper left body of the uterus (Fig 5). There was no free fluid or lymphadenopathy. Surgical removal of the uterus and the mass confirmed the diagnosis of a giant pedunculated subserosal leiomyoma with hyaline degeneration originating from a leiomyomatous uterus.

## Discussion

Leiomyoma (or fibroid) is the most frequently diagnosed gynecologic tumor, occurring in 20–30% of women older than 30 years. Leiomyomas arise from the overgrowth of smooth muscle and connective tissue of the uterus [1]. Histologically, a monoclonal proliferation of smooth muscle cells occurs.

The growth of a leiomyoma seems to depend on the hormone estrogen. As long as a woman with leiomyomas is menstruating, the leiomyomas will probably continue to grow, usually slowly [2].

Leiomyomas can undergo various types of degeneration as they enlarge. These include hyaline or myxoid degeneration, cystic degeneration, dystrophic calcification, and red degeneration [3]. Among them, hyalinization is the most common type of degeneration, occurring in up to 60% of cases [4]. Rarely, uterine leiomyoma may undergo malignant degeneration to become a sarcoma. The incidence of malignant degeneration is less than 1.0% and has been estimated to be as low as 0.2%.

According to their position within the uterine wall leiomyomas can be classified as a) intramural (70%), b) growing into the uterine cavity (10%) having either submucosal, pedunculated submucosal or pedunculated vaginal position or c) growing outwards from the uterus (20%) further classified as cervical, subserous, intraligamentous or pedunculated subserous (abdominal) fibroids [5]. Pedunculated uterine leiomyomas occur when the fibroid is in continuity with the uterus with a stalk and they may grow either within the uterine cavity (submucosal) or outside of the uterus (subserosal) simulating ovarian neoplasms [6]. They can become twisted and cause a kink obstructing blood vessels feeding the tumor that requires prompt surgery. The majority of uterine leiomyomas are confidently diagnosed sonographically. However, large, degenerated or atypical tumours-like in our case- may be a diagnostic challenge [7]. CT may help further characterize large pelvic and abdominal masses and determine their organ of origin, as in the present case [8]. In equivocal cases, magnetic resonance imaging is used as a problem solving tool to characterize uterine and adnexal pathology [9,10].

## Conclusion

As a conclusion, detecting the continuity of an abdominal mass with the uterus by a stalk on cross-sectional imaging -in the absence of accompanied ascites or elevated serum tumor markers- could lead to the diagnosis of a pedunculated subserosal leiomyoma.

## Consent

The authors confirm that informed written consent was received from the patient for publication of the manuscript and figures.

## Competing interests

The authors declare that they have no competing interests.

## Authors' contributions

AS made substantial contributions to analysis and interpretation of data and was a major contributor in writing the manuscript. AO made substantial contributions to conception and design and was a major contributor in writing the manuscript. KD made substantial contributions to acquisition, analysis and interpretation of data. PP revised the manuscript critically for important intellectual content. All authors read and approved the final manuscript.
